# Titanium implant surface roughness after different implantoplasty protocols: A laboratory study

**DOI:** 10.1002/cre2.659

**Published:** 2022-09-07

**Authors:** Hulya Yildiz, Kristina Bertl, Andreas Stavropoulos

**Affiliations:** ^1^ Department of Periodontology, Faculty of Dentistry Istanbul Aydın University Istanbul Turkey; ^2^ Division of Oral Surgery, University Clinic of Dentistry Medical University of Vienna Vienna Austria; ^3^ Department of Periodontology, Faculty of Odontology Univesity of Malmö Malmö Sweden; ^4^ Division of Conservative Dentistry and Periodontology, University Clinic of Dentistry Medical University of Vienna Vienna Austria

**Keywords:** diamond burs, implantoplasty, laboratory study, profilometer, surface roughness, tungsten carbide burs

## Abstract

**Objective:**

To compare the surface roughness of sandblasted, large grit, acid‐etched (SLA) surfaced titanium discs, after implantoplasty (IP) with different combinations of rotating instruments without or with the subsequent use of a silicone polisher.

**Methods:**

Titanium discs (*n* = 12 per group) with an SLA surface were treated with the following IP protocols: (1) Tungsten carbide bur sequence from company 1 (Komet Dental) without or with polishing (P) with a silicone polisher (Brownie®), (2) tungsten carbide bur sequence from company 2 (Hager & Meisinger GmbH) without or with P, and (3) diamond bur sequence (125, 40, 15‐μm grit) without or with P. Pristine turned (T) and SLA titanium discs were used as negative and positive controls, respectively. Surface roughness measurements were taken with a contact profilometer rendering *R*_*a*_ and *R*_*z*_ values.

**Results:**

All IP protocols, even without P, resulted in significantly reduced surface roughness compared to the SLA group. The tungsten carbide bur protocols, even without P, resulted in a surface roughness similar to or significantly lower than that in the T group in terms of *R*_*a*_ and *R*_*z*_, respectively. IP with the diamond bur sequence resulted in a significantly rougher surface compared to that achieved with the carbide burs. In all IP groups, P with a silicone polisher resulted in a significantly smoother surface.

**Conclusions:**

IP with dedicated tungsten carbide burs without or with the subsequent use of a silicone polisher resulted in a surface roughness similar to or significantly lower than that of commercially available turned surfaces. IP with a diamond bur sequence required additional polishing to achieve a comparable surface roughness to that of commercially available turned surfaces.

## 1 INTRODUCTION

Although dental implants show high survival rates (Jung et al., 2012; Pjetursson et al., 2012), mechanical or biological complications, occur in a high proportion of patients. For example, depending on the population examined and the level of bone destruction used for case definition, peri‐implantitis affects approximately from every fifth to the fourth patient (Derks & Tomasi, 2015) up to every second patient (Romandini et al., 2021). Treatment of peri‐implantitis remains a challenge and often includes surgery, ranging from open flap debridement to resective and reconstructive approaches (Figuero et al., 2014; Ramanauskaite et al., 2021; Renvert & Polyzois, 2015; Schwarz et al., 2022). In the case of implants with a modified (structured) implant surface, the surgical approach can be combined with implantoplasty (IP) (i.e., the mechanical modification of the implant surface, including the removal of the implant threads and smoothening of the implant surface, by means of rotating instruments), which—despite the favorable results presented in clinical studies (Bianchini et al., 2019; Englezos et al., 2018; Romeo et al., 2005, 2007)—remains a controversial procedure (Bertl & Stavropoulos, 2021; Stavropoulos et al., 2019).

IP is considered the only decontamination method that completely removes the biofilm (Bertl & Stavropoulos, 2021; El Chaar et al., 2020). Importantly, IP is supposed to improve soft tissue integration at the exposed implant surface and facilitate better/easier plaque removal (Azzola et al., 2020; Beheshti Maal et al., 2020; Schwarz et al., 2017). In this context, an implant surface with a mean *R*_*a*_ (arithmetic mean roughness) <0.2 μm is considered a clinically acceptable “threshold” value in terms of microbial colonization (Bollen et al., 1997; Quirynen et al., 1996; Teughels et al., 2006). Previous laboratory studies assessing surface characteristics after IP have reported *R*_*a*_ and *R*_*z*_ (averaged roughness) values ranging from 0.32 to 0.98 μm and from 1.87 to 6.86 μm, respectively (Ramel et al., 2016; Sahrmann et al., 2019). The results of IP in terms of surface roughness, in those reports, were strongly dependent on the type and sequence of burs and/or polishers used.

The objective of the present laboratory study was to compare the surface roughness of sandblasted, large grit, acid‐etched (SLA) surfaced titanium discs, after IP with different combinations of tungsten carbide or diamonds burs, without or with the subsequent use of a silicone polisher (P).

## 2 MATERIALS AND METHODS

### 2.1 Titanium discs

For the present laboratory study, 60 grade IV titanium discs with a diameter and thickness of 10 and 2.5 mm, respectively, were used. Forty‐eight discs had an SLA surface (Trias‐ixx2, Servo Dental GmbH & Co. KG, Germany), while 12 discs had a turned surface.

### 2.2 IP procedure

**Figure 1:**
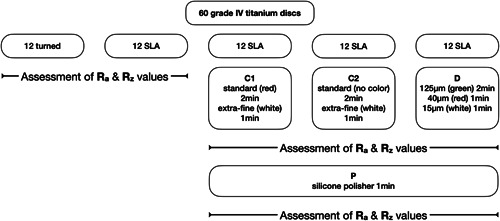
Study outline

The 12 discs with a turned surface were considered a negative control group, while 12 discs with an SLA surface served as the positive control group (i.e., were not subjected to IP). The remaining 36 discs with SLA surfaces were divided into three equally sized groups and were subjected to different IP protocols using tungsten carbide and diamond burs. Specifically, the following IP protocols were assessed: (1) C1: 2 tungsten carbide burs with standard (red ring) and extra‐fine (white ring) toothing (Komet Dental); (2) C2: 2 tungsten carbide burs with standard (no color) and extra‐fine (white ring) toothing (Hager & Meisinger GmbH); and (3) D: 3 diamond burs with decreasing grit (125 [green ring], 40 [red ring], and 15 μm [white ring]) (Komet Dental). The standard tungsten carbide burs and the green diamond burs were used for 2 min, while the extra‐fine tungsten carbide burs and the red and white diamond burs were used for 1 min each. After assessment of the surface roughness the same 36 discs were polished with a low‐speed handpiece at 15.000 rpm (W&H) with a silicone polisher (Brownie®; Shofu Dental GmbH) for 1min (P), that is, C1 + P, C2 + P, D + P (Figure 1).

### 2.3 Assessment of the surface roughness

The surface roughness was analyzed with a mobile two‐dimensional contact stylus profilometer (MahrSurf M 400, Mahr GmbH). The surface roughness of all samples was measured in a straight line at a constant speed and pressure. The measuring length was 5.6 mm using a cut‐off of 0.8 mm. *R*_*a*_ and *R*_*z*_ values were provided for each disc (ISO 12085). *R*_*a*_ represents the mean of the absolute values of the modified roughness profile, based on the central line to a reference route, and *R*_*z*_ represents the arithmetic mean of the differences between the five highest and five lowest points of a profile within a sample route on the measured surface.

### 2.4 Statistical analysis

The surface roughness values (i.e., *R*_*a*_ and *R*_*z*_ values) were not normally distributed, as confirmed by the Q–Q plots and Shapiro–Wilk test. Nonparametric tests were thus applied: (1) For comparison of the negative and positive control group and the three IP protocols without P (i.e., five groups in total) Kruskal–Wallis H‐Test with Mann–Whitney U‐test as post‐hoc test was applied, and (2) for the comparison before and after P Wilcoxon–Signed Rank Test was applied due to dependency of the data. Stata 17.0 was used for statistical analysis and *p*‐values ≤ 0.05 were considered as statistically significant.

## 3 RESULTS

### 3.1 Surface roughness after IP with tungsten carbide or diamond burs

**Figure 2:**
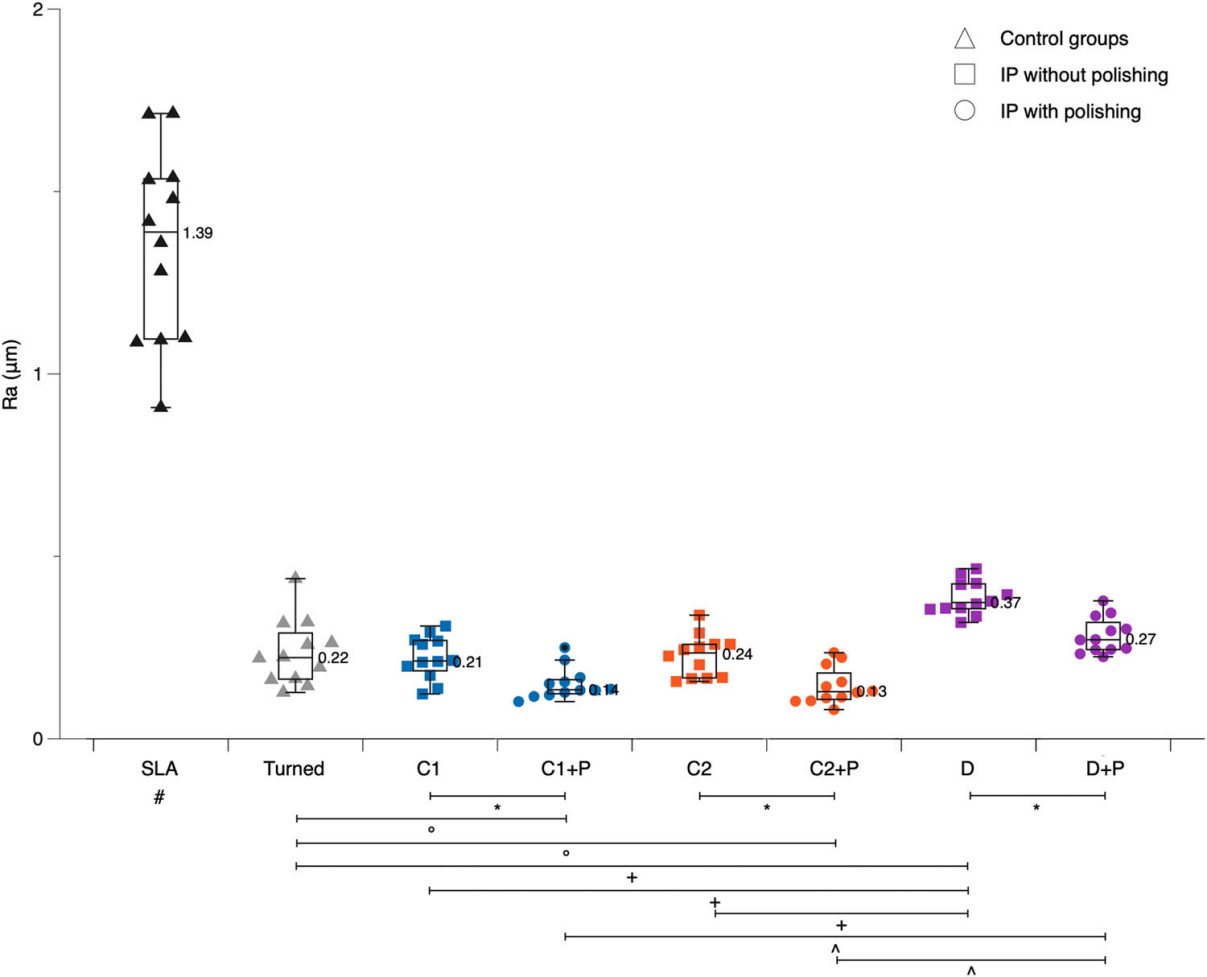
Boxplots presenting individual and median *R*_*a*_ values of the various control and test groups before and after polishing (P) with a silicone polisher. # SLA displayed significantly higher values than all other groups (*p* < .001); *P resulted in significantly lower values (*p* < .01); ° turned surface displayed significantly higher values (*p* < .01); + D displayed significantly higher values (*p* < .001); ^ D + P displayed significantly higher values (*p* < .001).

**Figure 3:**
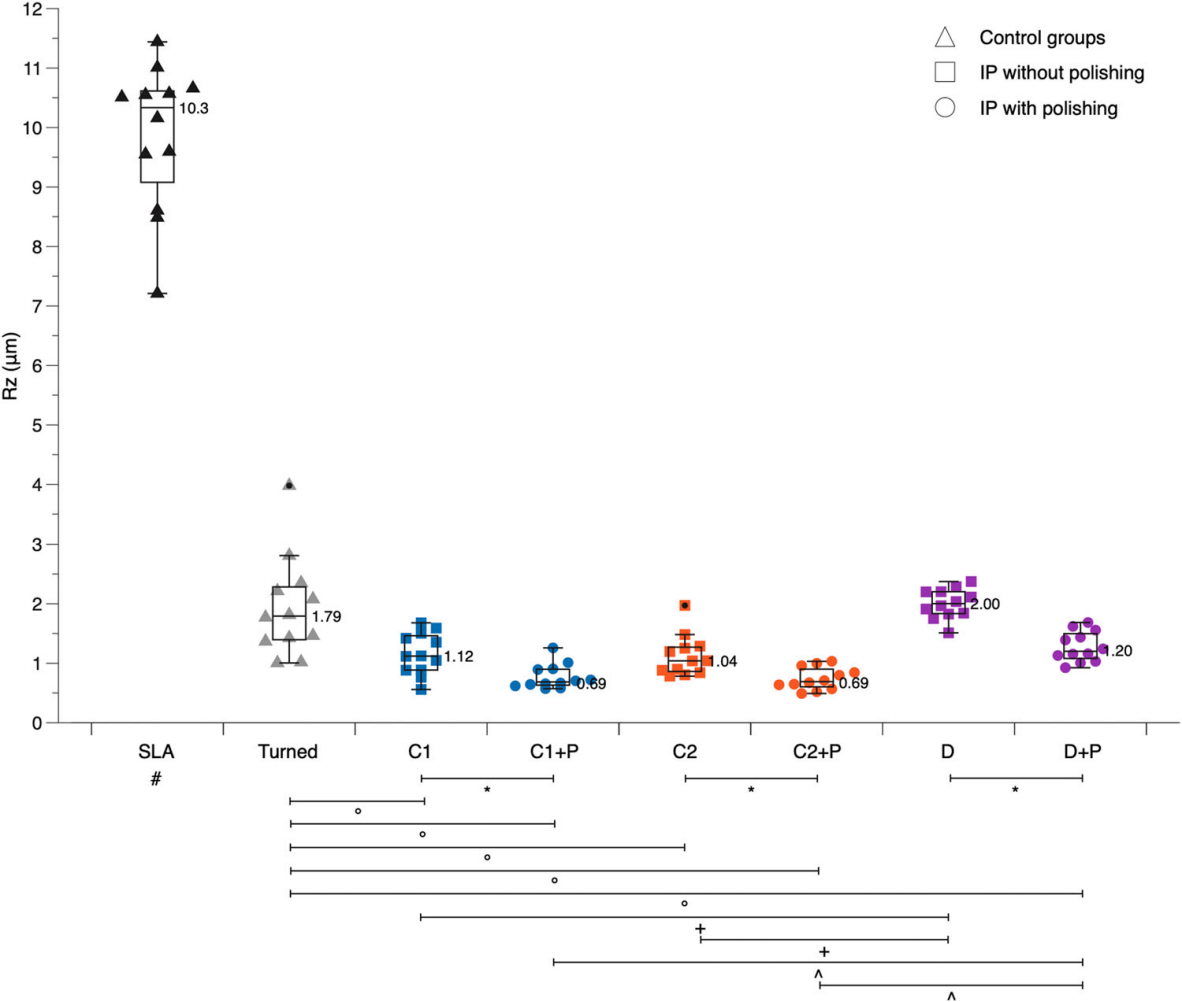
Boxplots presenting individual and median *R*_*z*_ values of the various control and test groups before and after polishing (P) with a silicone polisher. # SLA displayed significantly higher values than all other groups (*p* < .001); *P resulted in significantly lower values (*p* < .01); ° turned surface displayed significantly higher values (*p* < .05); + D displayed significantly higher values (*p* < .001); ^ D + P displayed significantly higher values (*p* < .001).

The median *R*_*a*_ and *R*_*z*_ values ranged after IP with tungsten carbide or diamond burs from 0.21 to 0.37 μm (Figure 2) and from 1.12 to 2.00 μm, respectively (Figure 3). In comparison the turned and SLA surfaces showed median *R*_*a*_ values of 0.22 and 1.39 μm, respectively, and median *R*_*z*_ values of 1.79 and 10.3 μm, respectively. As expected, the pristine SLA discs displayed a significantly rougher surface compared to all other groups (*p* < .001).

In terms of *R*_*a*_ values, IP with tungsten carbide burs, irrespective of manufacturer, resulted in a similar surface roughness compared to the turned surface (C1: *p* = .887; C2: *p* = .854), while the diamond bur combination (D) remained significantly rougher than the turned surface (*p* < .001). There was no significant difference between the two different tungsten carbide bur sets (*p* = .932), but both were significantly smoother than the diamond bur sequence (C1: *p* < .001; C2: *p* < .001) (Figure 2).

In terms of *R*_*z*_ values, IP with tungsten carbide burs, irrespective of the manufacturer, resulted in a significantly smoother surface compared to the turned surface (C1: *p* = .007; C2: *p* = .003), while the diamond bur combination (D) was comparable to the turned surface (*p* = .378). There was no significant difference between the two tungsten carbide bur sets (*p* = .590), but both were significantly smoother than the diamond bur sequence (C1: *p* < .001; C2: *p* < .001) (Figure 3).

### 3.2 Surface roughness after IP with tungsten carbide or diamond burs and subsequent P

The median *R*_*a*_ and *R*_*z*_ values ranged after IP with tungsten carbide or diamond burs and subsequent P with a silicone polisher from 0.13 to 0.27 μm (Figure 2) and from 0.69 to 1.20 μm, respectively (Figure 3). In all three groups (i.e., C1, C2, and D) P significantly smoothened the surface (*p* < .01).

In terms of *R*_*a*_ values, IP with tungsten carbide burs, irrespective manufacturer, and subsequent P resulted in a significantly smoother surface compared to the turned surface (C1 + P: *p* = .004; C2 + P: *p* = .003), while the diamond bur combination achieved after P (D + P) a comparable surface roughness to the turned surface (*p* = .068). Further, there was no significant difference between the two different tungsten carbide bur sets after P (*p* = .469), but both were significantly smoother than the diamond bur sequence even after P (C1 + P: *p* = .005; C2 + P: *p* = .005) (Figure 2).

In terms of *R*_*z*_ values, all three test groups were after P significantly smoother than the turned surface (C1 + P: *p* < .001; C2 + P: *p* < .001; D + P: *p* = .024). Also, after P there was no significant difference between the two tungsten carbide bur sets (*p* = .799), but both were significantly smoother than the diamond bur sequence after P (C1 + P: *p* = .007; C2 + P: *p* = .002) (Figure 3).

## 4 DISCUSSION

The result of the present study showed that IP with a diamond bur sequence resulted in a rougher surface compared to that achieved with tungsten carbide burs, while with additional polishing, a surface roughness to that of commercially available turned surfaces was achieved. Furthermore, IP with dedicated tungsten carbide burs without or with the subsequent use of a silicone polisher resulted in a surface roughness similar to or significantly lower than that of commercially available turned surfaces.

These results are in accordance with those presented in a quite recent systematic review (Burgueño‐Barris et al., 2021), summarizing the available literature on surface roughness after IP with different drilling protocols. In this review, the combination of tungsten carbide burs and silicone polishers was reported to deliver the best results (i.e., smoothest surface). Nevertheless, the lowest *R*_*a*_ and *R*_*z*_ values reported in the individual studies included in this systematic review (*R*_*a*_ approximately 0.3–0.4 μm; *R*_*z*_ approximately 1.9–2.3 μm) were slightly higher than what was achieved herein (*R*_*a*_ < 0.2 and *R*_*z*_ < 1.0 μm). This slight variation in terms of surface roughness values can be attributed to differences in the method of performing IP, for example, variable rpm, standardized versus unstandardized applied pressure, variable predefined timeframes versus subjective visual assessment of smoothness, and so forth.

As mentioned earlier, IP primarily aims in creating a surface that is less conducive to and/or facilitates easier plaque removal, thereby enhancing soft tissue integration on the exposed implant surface and, more importantly, reducing the risk for disease recurrence (Beheshti Maal et al., 2020; Schwarz et al., 2017). Surface roughness characteristics are relevant in terms of biofilm growth/regrowth and an *R*_*a*_ value of 0.20 µm has been previously considered a clinically acceptable “threshold” since higher surface roughness values have been associated with increased plaque formation (Bollen et al., 1997; Quirynen et al., 1996; Teughels et al., 2006). Herein, IP only with tungsten carbide burs resulted in *R*_*a*_ values close to 0.20 µm, representing thus a rather favorable outcome and making thus the use of silicon polishers obsolete. Indeed, the results of a recent, proof‐of‐principle study (Azzola et al., 2020), seem to support the notion that an implant surface after implantoplasty collects less plaque than a pristine moderately rough implant surface. In this study, a splint with three implants with a moderately rough surface and three implants subjected to IP was worn by a single volunteer for a period of 5 days. Scanning electron microscopy analysis showed that about 65% of the surface of the moderately rough implants versus only 16% of the surface of those implants subjected to IP was covered with plaque (Azzola et al., 2020). Furthermore, the biocompatibility of the titanium surface generated from IP has been shown in various studies. For example, in in vitro studies, cell viability of gingival fibroblasts (Schwarz et al., 2017) or of osteoblast‐like‐cells (Toma et al., 2016) was not affected negatively by IP, while in another recent in vitro study, enhanced gingival fibroblast growth was observed on smoother surfaces versus rougher surfaces post‐IP (Beheshti Maal et al., 2020).

In this context, several limitations and potential complications are discussed in regard to IP, for example, reduced implant strength and/or titanium and silicone particle deposition in the surrounding tissue after IP. For example, a recent laboratory study demonstrated that IP significantly reduced the maximum implant failure strength, irrespective of implant type/design, diameter, or material, but in general, the maximum implant failure strength remained high (Bertl et al., 2021). However, in a recent systematic review that summarized all laboratory, preclinical in vivo, and clinical research on IP (Stavropoulos et al., 2019), no remarkable mechanical or biological complications associated with IP, in the short‐ to medium‐term, were identified. Only a single case of mucosal tattoo due to the titanium particle debris generated during IP was reported in one of the studies included in this review. Indeed, the deposition of a certain amount of titanium particles in the neighboring tissues during IP is hardly preventable. The number, size, and composition of the titanium particles generated from IP seem dependent not only on the implant producer and material (Barrak et al., 2020; Wu et al., 2022) but also on the type of rotating instruments used (Beheshti Maal et al., 2020). The relevance of titanium particle deposition within the neighboring tissues due to IP is not completely understood, and it has been suggested that the presence of titanium particles within the peri‐implant tissues may play a negative role in the pathogenesis of peri‐implantitis (Fretwurst et al., 2018). Nevertheless, as shown very recently, titanium particles are present in the granulation tissue surrounding an implant affected by peri‐implantitis, but histopathological analysis did not indicate any direct pathological effects and/or a marked biological response due to these titanium particles (Rakic et al., 2022). In perspective, the reduction of debris from IP should be considered beneficial. Thus, a relevant finding herein is that additional polishing with silicon polishers, to achieve a surface comparable to commercially available turned implant surfaces and to roughly approximate the above‐discussed threshold of *R*_*a*_ values of 0.20 µm, is not necessary when using dedicated tungsten carbide burs.

The present laboratory study comes with an inevitable limitation, that is, using discs—instead of implants—allows most likely for higher standardization of the procedure. In contrast, in the clinic, the outcome of IP is much dependent on proper access to the implant surface so that the burs are parallel to the implant axis; this, in turn, depends on the possibility to remove the prosthetic restoration during the surgical procedure, on implant macrodesign characteristics (e.g., bone vs. tissue level implants), on the morphology of the bone defect, and/or on the location in the mouth. Thus, it might be challenging to achieve a similarly smooth surface in the clinic, as it was achieved herein. Furthermore, in this study, the thickness of the discs before and after IP was not recorded. Thus, differences in the actual amount of metal removed with the various procedures tested herein cannot be excluded.

In conclusion, the results of the present laboratory study (1) confirmed the previously reported superiority of tungsten carbide burs over diamond burs in terms of implant surface roughness after IP, and (2) indicated that IP with dedicated tungsten carbide burs, irrespective of the manufacturer, resulted in a surface roughness similar to or significantly lower than that of commercially available turned surfaces, and thus use of silicon polishers seems obsolete.

## AUTHOR CONTRIBUTIONS

**Hulya Yildiz**: Conceptualization; execution; assessment; interpretation; manuscript drafting. **Kristina Bertl**: Conceptualization; analysis; interpretation; manuscript drafting. **Andreas Stavropoulos**: Conceptualization; interpretation; manuscript drafting.

## CONFLICT OF INTEREST

The authors declare no conflict of interest.

## Data Availability

The data are available from the authors upon reasonable request.
